# Fingerprinting metal transfer from mantle

**DOI:** 10.1038/s41467-019-11445-w

**Published:** 2019-08-05

**Authors:** Zengqian Hou, Rui Wang

**Affiliations:** 1 Institute of Geology, Chinese Academy of Geological Sciences (CAGS), 100037 Beijing, China; 2 State Key Laboratory of Geological Processes and Mineral Resources, and Institute of Earth Sciences, China University of Geosciences, 100083 Beijing, China

**Keywords:** Geochemistry, Petrology

## Abstract

The ore-forming magmas in post-subduction copper deposits are thought to be derived from the lower crust. The Au-Te fingerprints of post-subduction magmas reveal an important role for the metasomatized sub-crustal lithospheric mantle in the formation of porphyry and epithermal copper deposits.

Cu, Au, Mo, and platinum group elements (PGE) have relatively low concentrations in the Earth’s crust and mantle compared with that in magma-related ore deposits, such as porphyry and epithermal deposits with 1000 to 10,000 times of metal enrichment. Magma genesis and evolution are thought to play the crucial role in metal transport and enrichment. Holwell et al.^1^ proposes a new insight that links the formation of porphyry and epithermal deposits with magmas originated from lithospheric mantle in post-subduction setting. Porphyry and epithermal deposits are the major source of Cu, Mo, and Au, and significant source of Ag, Sn, W, and rare-earth elements^2^. Porphyries can occur in subduction zones, and also post-subduction settings. However, it has been unclear whether the metals of porphyry and epithermal mineral systems are derived from mantle or crust in post-subduction settings. Writing in *Nature Communications*, Holwell et al.^1^ argue that Te is an excellent tracer of sub-crust lithospheric source to track its fluxing of metals into the crust that has not previously been used.

The major chalcophile elements of ores, such as Cu, Mo, and Au, are mainly from magmatic fluids exsolved from fertile magmas^3^. In island arcs and continental arcs, where porphyry and epithermal deposits form, it is generally thought that oxidized, S-, and Cl-rich fluids released from subducting slabs migrate into the asthenospheric mantle wedge, where they cause partial melting and mobilization of metals^4^, and ultimately transfer these metals into the crust (Fig. 1a). The characteristics of post-subduction porphyry and epithermal deposits are similar in many aspects to those in arc settings, i.e., mineralization style, alteration zoning, and metal association^5^. However, their petrogenesis and tectonic controls are different. The Gangdese belt in southern Tibet, with Cu resources over 25 Mt, is ranked as the most representative geological domain for post-subduction mineral deposit^6^. The Cenozoic post-subduction porphyry Cu–Mo deposits are widely developed in the eastern Gangdese belt, and include the giant Qulong and Jiama deposits^5^. The ore-forming magmas have distinctively higher La/Yb and Sr/Y ratios compared with normal arc rocks in the Gangdese belt, but many other major and trace elements overlap with the felsic endmembers of arc magmas^6^. Their Sr–Nd–Hf isotope compositions are very similar to the cospatial arc magmas^7^. These features suggest that the post-subduction granitoids formed by melting of the previous arc root. The exposed Gangdese arc root^8^ after granulitization detailed further the existence of such lower arc crust with Cu enrichment, which is able to generate ore-forming porphyries in experimental melting^9^.

**Fig. 1:**
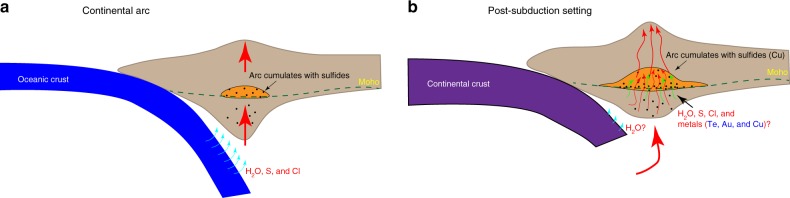
Petrogenesis of ore-forming magmas in post-subduction setting. **a** During continental arc subduction, dehydration of the subducting oceanic crust leads to hydration of the overlying mantle and then partial melting of asthenospheric mantle wedge. Hydrous basaltic melts intrude the overlying lithosphere and pool at the base of the crust, where they fractionate and generate arc cumulates with sulfides. More evolved, less dense magmas rise to the upper crustal levels. **b** Underplating and crystallization of previous arc magmas in the lower crust form hydrous cumulates, in which accumulation of Cu sulfides depends on redox state of arc magmas. Relatively low-oxidation state of previous arc leads to Cu enrichment in cumulates, thus providing metal source for younger, post-subduction-related porphyry and epithermal systems. Upwelling of asthenospheric mantle related to slab tearing or breaking off triggers remelting of Cu sulfide-bearing cumulates, leading to formation of fertile magmas for post-collisional Cu deposit formation. Mantle upwelling can also induce small volumes of melts and fluids from subcontinental lithospheric mantle (SCLM), which might trigger fluxes of arc cumulates with input of H_2_O, S, Cl, and possibility metals of Te, Au, and Cu, which are essential for formation of porphyry and epithermal deposits

With the absence of subducted oceanic crust and sediments to provide H_2_O, S, Cl, and high-oxidation state in post-subduction settings, the genesis of giant porphyry deposits remains controversial. In agreement with Holwell et al.^1^, the input of hydrous alkaline-rich mantle melts from the post-subduction metasomatised lithospheric mantle was an important requirement for the formation of hydrous and fertile magmas in the Gangdese belt. The Miocene high-Sr/Y granitoids in this belt yield temperatures that are generally <750 °C^6^. Given that dehydration melting of the crust requires temperatures of at least 850 °C to generate reasonable granitic magma volumes, the consistently low temperatures for the Miocene high-Sr/Y granitoids requires additional water-fluxed melting^10^. Lithospheric mantle-derived trachytic magmas, that are coeval with high-Sr/Y granitoids, likely released water during ascending to stimulate crustal melting^6,11^. Deep crustal and mantle xenoliths entrained by the Miocene trachytes provide direct information regarding crust-mantle hybridization^12^, and suggest a link between the origin of high-Sr/Y granitoids and the coeval Miocene trachytic volcanic rocks. This is also evident in their slightly higher CaO, MgO, Ni, and Zr contents and lower εNdi for granitoids which contain trachytic magmatic component^2^. In addition to water input, volatile-rich trachytic melt might also transfer S and Cl into the crust (Fig. 1b). These H_2_O, S, and Cl components are essential for the porphyry and epithermal deposit formation^13^.

Holwell et al.^1^ propose that an important signal of metal transfer from the mantle to the crust is provided by their chalcophile and PGE composition, in particular Ni–Au–Te contents in the igneous rocks associated with porphyry and epithermal deposits in support of metasomatized subcontinental lithospheric mantle (SCLM) source model. While we fully appreciate the innovation of this proposed process, we notice, however, that the Au–Te compositions of the lower crust and metasomatized SCLM are indistinctive. Alternatively, the subduction-modified lower crust has been proved to be metal (such as Cu and Au)-rich, and its melt has the capability to form large porphyry and epithermal deposits^14,15^. The middle–lower crust in post-subduction settings has recently been studied for sulfide compositions, and most of them (such as Kohistan arc lower crust outcrops, Gangdese arc cumulates, Yunnan lower-crust garnet amphibolite xenoliths) are Cu-rich (up to 1000 ppm^16^) and Au-rich (up to 16 ppb^15^). These ore-forming components (Au, Cu), though locally remobilized during later metamorphism, could be preserved in arc cumulates in the lower continental crust (Fig. 1). The incubation time between crustal base metal enrichment and reactivation can be short in a successive process from subduction to collision, such as in the Gangdese porphyry deposits^2^, or it can be delayed until later reactivation of the cratonic margin in Yunnan^15^. The metals could be entrained by later magmas or fluids ascending upward^17^. The evidence of fluxing metals from SCLM melts to post-subduction high-Sr/Y melts to make the system fertile is still missing.

Holwell et al. approach^1^ is inspiring toward understanding this metal conundrum from the mantle or the crust. There is still work we can do to explore more fingerprints to identify exactly what the metal sources are and how they operate. For examples, the metals, their own isotopes, such as Cu^18^, Zn, and Fe, may provide direct constraints on their sources. In addition, experimental melting of lower crust and sub-crustal mantle materials may provide significant implication for metal transportation and deposit formation.
